# Viticultural and Pre-Fermentation Strategies to Reduce Alcohol Levels in Wines

**DOI:** 10.3390/foods14152647

**Published:** 2025-07-28

**Authors:** Francesca Coppola, Bruno Testa, Mariantonietta Succi, Gianluca Paventi, Catello Di Martino, Massimo Iorizzo

**Affiliations:** 1 Department of Agricultural Sciences, University of Naples “Federico II”, 80055 Portici, Italy; francesca.coppola2@unina.it; 2 Department of Agricultural, Environmental and Food Sciences, University of Molise, Via De Sanctis, 86100 Campobasso, Italy; bruno.testa@unimol.it (B.T.); lello.dimartino@unimol.it (C.D.M.); iorizzo@unimol.it (M.I.)

**Keywords:** alcoholic beverages, alcoholic fermentation, innovative winemaking, ethanol

## Abstract

Changes in lifestyles, as well as the growing attention to healthy nutrition, led to the increasing demand for wines with reduced alcohol content. The reduction in fermentable sugars in the pre-fermentation stage of wine is one of the common methods for the production of wines with lower alcohol content. Viticultural practices such as early harvesting, use of growth regulators, reducing leaf area to limit photosynthetic rate, and pre-harvest irrigation are utilized. Additionally, techniques such as juice dilution, juice filtration with membranes, and the use of enzymes (e.g., glucose oxidase) are also employed in the pre-fermentation stage. This review summarizes and describes the classic and innovative viticultural and pre-fermentation techniques used to reduce the alcohol content and their main impact on the compositional characteristics of wine.

## 1. Introduction

The global market for both low- and zero-alcohol beverages is undergoing significant global expansion, growing by 34% between 2020 and 2023, and the International Wine and Spirits Research (ISWR) predicts that the Compound Annual Growth Rate will grow by +6% between 2023 and 2027 with an increase in +7% in the non-alcohol category and +3% in the low-alcohol category [1,2,3]. With health risks awareness, the predilections of consumers are shifting toward new product offerings, alternatives to traditional alcoholic beverages, with an increasing percentage of the adult population seeking more frequently lower alcohol wines [4]. In addition, these products meet the needs of those who avoid alcohol for safety reasons, such as those who have to drive, as well as for reasons related to age or religious beliefs [5,6]. Finally, some consumers are attracted to new trends and want to try innovative and trendy consumer experiences [7,8,9,10,11]. Although this represents an expanding industry, it must also be noted that many consumers are still reluctant to buy or consume wines with a low alcohol content, due to their taste and/or their price, often the same as traditional wine [11,12,13].

However, the use of alcoholic beverages and the related health effects are a global problem and therefore need to be addressed not only by individual nations but also on an international level. The World Health Organization (WHO) leads a global strategy to reduce the harmful use of alcohol worldwide [14].

In the winemaking sector, some countries present a complex scenario about laws dealing with no- and low-alcohol wine due to the occurrence of different government authorities legislating on this aspect. As an example, the Federal Alcohol Administration Act (FAA Act) of the United States reports an alcohol content ranging from 7% to 24% *v*/*v* alcohol for wine. Dealcoholized wines containing less than 7% *v*/*v* alcohol are not regulated by the FAA Act, leaving them to the labeling instructions of the Federal Food, Drug, and Cosmetic Act (FD&C Act) [15].

By contrast, the Australian Food Standards Code recognizes as “low alcohol” only the beverages with less than 1.15% (*v*/*v*) alcohol, whereas it does not define the word “dealcoholized” [16]. These products are often labeled with statements such as “dealcoholized wine”, “less than 0.5% *v*/*v* alcohol”, and “less than 1.0% *v*/*v* alcohol”. For beverages containing less than 0.5% *v*/*v* alcohol is not mandatory to report the alcohol content; however, many producers voluntarily add the information on the label [17].

In Europe, the sector is governed by Regulation (EU) 1308/2013 supplemented by Regulation (EU) 2021/2117, which establishes the definition and classification of dealcoholized wines (alcohol content not exceeding 0.5%) and partially dealcoholized wines (alcohol content higher than 0.5% but lower than the minimum of the original category), as well as the technologies (partial evaporation under vacuum, membrane filtration, and distillation techniques) that can be adopted to obtain the production of totally and partially dealcoholized wines [18,19]. In defining this new framework, the EU has used the recommendations by the International Organization of Vine and Wine (OIV) as a basis, in particular Resolutions OIV-ECO 433-2012, OIV-ECO 432-2012, OIV-OENO 394A-2012, and OIV-ECO 523-2016 [20,21,22,23].

Besides legal aspects, the great demand for alcohol-free and/or low alcohol beverages represents a great challenge for the production of wines with controlled alcohol content by using sustainable practices [10], even more in light of the progressive increasing temperature due to climate change [24]. Therefore, winemakers are really seeking different possibilities at the various stages of winemaking for obtaining high-quality wines with a reduced alcohol content [25]. At present, strategies for reducing wine alcohol content can be classified into pre-fermentation, fermentation, and post-fermentation techniques [26].

Wine alcohol content reduction can be achieved by acting at three different stages, the pre-fermentation, fermentation, and post-fermentation stages, based, respectively, on the decrease in fermentable sugars, the reduction in alcohol production, and the removal of produced ethanol [25,27,28,29]. However, the technological procedures used during the first two stages usually result in wines with a reduced alcohol content, but still remaining above the required ethanol concentration needed to fall under dealcoholized wines. By contrast, post-fermentation strategies, namely distillation-based, membrane-based techniques, and their combination, have been used for the removal of alcohol up to a content lower than 0.5% *v*/*v* [30].

For several wines, a remarkable demand for the reduction in alcoholic strength has been progressively growing, as well as there is also an increasing interest in the production of wines with low and/or zero alcohol content able to suit market requirements, but at the same time, to preserve their sensory qualities.

This review summarizes and describes the classic and innovative viticultural and pre-fermentation techniques used to reduce the alcohol content and their main impact on the compositional characteristics of wine.

## 2. Agronomic Strategies

The development of viticultural practices, in most cases, has historically been aimed at promoting the accumulation of sugars in the berries [31]. Today, in the context of global warming, physiology of grapevines is changing; these modifications exert a profound shift in both primary and secondary metabolisms in berry, responsible for the balance of sugar and organic acids (the former) and the production of phenolic and aromatic compounds (the latter), which together result in wine composition [24,32].

Therefore, with a view to producing wines with a low alcohol content, the concept of grape ripening must be reconsidered and must necessarily include other parameters such as polyphenols, aromas, and acidity/alcohol balance in addition to sugar level [33,34,35,36,37].

About these latter, a clearer knowledge of the factors influencing grape sugar content represents a key aspect, since it allows for selecting the best harvesting time, as well as coping with potential adaptations related to climate change. In this regard, it must be noted that the wine production has been strongly affected by recent climatic trends, since the increased temperatures resulted in higher sugar levels at harvesting, as well as a shortened and early season shifted ripening period [38]. The prediction of the progressive global warming along this century makes mandatory the full understanding of whether and how environmental factors could affect grape berry ripening dynamics in order to better plan adaptation strategies [39]. Therefore, winegrowers and winemakers are required to adopt cultivation approaches and strategies resulting in grapes without excessive sugar concentrations, but preserving a satisfactory phenolic and/or aromatic maturity [29,40].

In viticulture, three strategies can basically affect alcohol level in wines; these approaches are very different from each other and can be summarized as follows: (i) choice of grape variety; (ii) location of the vineyard; (iii) management practices [41,42].

Among these approaches, the latter appears the most interesting since, differently from the others, it does not require vineyard substitution and is directly appliable on existing vineyards. Considering the physiological mechanism of the plant, winemakers can employ different viticultural practices useful to modulate vine growth, vine development, and fruit ripening in consideration of environment conditions: these are canopy management, pre-harvest irrigation, and early grape harvest (Table 1). These viticultural strategies should be taken into account to improve the adaptation of vineyards and their ripening in a warmer climate and contribute to the reduction in alcohol in wines [24].

For instance, the increase in irrigation regimes improves vine water status and has been proven to stimulate both vegetative growth and yield, leading to a delay in the harvest date as a frequent consequence [64]. By contrast, the practice of defoliation, by reducing the vine’s source capacity to produce carbohydrates, has been reported to result in reduced concentrations of grape sugar [65].

### 2.1. Canopy Management

Although it is possible to lower wine alcohol content post-fermentation at present, it would be more useful to start the process in the vineyard. In this regard, proper grapevine canopy management can also be used to control the accumulation of sugar during grape ripening, resulting in wines with a low alcohol content [66]. In addition, several viticultural techniques such as severe shoot trimming, minimal pruning, late winter pruning, and leaf removal may delay grapevine ripening [41].

#### 2.1.1. Defoliation, Trimming, and Topping

Defoliation, otherwise referred to as “leaf removal” or “leaf thinning”, is a widely used practice in vineyard canopy management for the productivity and quality of grapes [65,67,68,69]. The primary objective of early leaf removal practices is to promote both aeration and drying in order to mitigate yield loss due to diseases, such as gray mold (*Botrytis cinerea*) and sour rot, particularly in compacted cluster varieties [70,71]. Another main purpose of early leaf removal is to enhance fruit and wine quality in a cool climate. In addition, defoliation at different canopy positions using various methods (e.g., manual or mechanical defoliation practices) leads to a reduction in the photosynthetic active leaf area (LA) [72]. It is known, in fact, that sugar accumulation rate in berries is largely dependent on the ratio of LA to fruit weight (FW), and LA reduction strongly affects this rate, which can result in wine with a decreased alcohol content [73].

The effects of defoliation on the composition of the harvested grapes and related wines have variable results depending on several factors, among which the main ones are cultivars and clones, timing, methods (mechanical or manual), environmental conditions, defoliation site (basal or apical leaf removal) [43,67,74,75,76,77].

Many studies reported that grapes of defoliated vines generally show higher sugar content and lower titratable acidity, besides affected flavonoids and phenolic total content, with respect to grapes from non-defoliated vines [67,74,78,79].

Otherwise, several reports showed that defoliation, by decreasing the formation of carbohydrates in the growing season, might result in a negative effect on subsequent grapevine productivity [80,81]. However, in other studies, the delay in berry ripening was obtained by decreasing the ratio between LA and yield (defoliation), resulting in a reduced concentration of fermentable sugars in grapes during ripening, with no negative impact on the capacity of the vine in the following year [82,83].

Another study reported that the late leaf removal above a Sangiovese bunch area (at post veraison, average 12 °Brix) resulted in a significant reduction in the content of total soluble solids in grape must, but did not significantly affect different compositional parameters such as phenolic substances [69]. Similar results were also very recently found for the Pinot noir variety [84].

By comparing apical and basal defoliation, Zhang and coauthors showed that the former approach limited wine alcohol content without severely affecting aromatic properties of wine, thus suggesting apical defoliation as a candidate for contrasting the increase in wine alcohol content resulting from global warming effects [44]. In this regard, it must be noted that the zone of defoliation also presents a remarkable influence since apical defoliation results in effects quite similar to shoot trimming (see below).

It is therefore necessary to carefully evaluate the effects of this practice on the vegetative-productive balance of the vine and on the qualitative characteristics of the grape [85].

Although defoliation practices result in ripening delay, they could also lead to excessive bunch exposure and, therefore, heat-damaged fruit. In addition, unbalanced vines, the ones in which the vegetative vigor is too high compared to the fruit load, could cause an imbalance in grape composition. In this regard, besides the zone, also the technique and phenological phase in which defoliation takes place greatly influence both sugar concentration and pH, as well as the phenolic profile and the aromatic component of the harvested grapes [75,79,86,87,88,89,90,91].

Accordingly, it has been found that late leaf removal (post-veraison defoliation) causes a reduction in the concentration of soluble solids in the juice (−0.7 °Brix compared to control), but negatively influences anthocyanin and phenolic compounds levels in Montepulciano grapevines [92].

A concomitant decrease in both grape anthocyanin accumulation and berry sugar concentration has been reported as a result of late leaf removal in Bobal and Tempranillo vines in eastern Spain [93].

In a recent study, *Vitis vinifera* L. cv. Grenache has been trained in an open-vase system in La Rioja (Spain); at two different stages (after fruit set and at veraison), two severe leaf removals were carried out and compared with each other, besides the control (untreated). Both leaf removal treatments tended to decrease sugar content with no effect on yield; these effects were highly affected by the year. Defoliation accounted for a decreased flavanol and stilbene content in berries at harvest [75].

In another study, the impact of defoliation at three different phases: pre-flowering (1), post fruit set (2), and at veraison (3), was investigated on yield components, grape and wine composition of the cv. Trnjak. Defoliation activities in 1 and 3 resulted in the lowest (19.5 °Brix) and highest (22.3 °Brix) level for grape sugar, respectively. Treatments 1 and 2 also gave wines significantly reduced in their ethanol content (about 12% *v*/*v*) in comparison to the wine obtained by treatment 3 (about 14% *v*/*v*). Defoliation at a later stage (3) also resulted in reduced levels of anthocyanins and flavonols compared to those registered for control wine [45].

Post-veraison defoliation in the upper middle part of the canopy of Sangiovese vineyard resulted in a reduced content of soluble solids in the berries and a lower alcohol wine, with minimal changes in anthocyanins and phenolics [43].

Torres and coauthors revealed that in warm climates, actions in canopy management need to be reviewed in the light of climate change. In regions affected by warm climates, the high temperature and the increased solar radiation did not result in higher wine quality, due to enhanced cluster exposure [94].

On the basis of the above-mentioned studies, significant research is still needed to determine the optimal ratio of leaves to crops, the timing and location of leaf removal from vines relative to fruit location, and the long-term impact on vine physiology [83,95].

The identification of updated climatic reports and phenology of the vine could be an important step to optimize the defoliation technique with a view to climatic changes and to reduce the sugar content of the grapes without penalizing other components of the grapes.

Shoot topping (ST), also known as tipping, is a typical viticulture practice in which the shoot tip is removed in order to limit excessive growth [95], thus removing a consistent sink for nutrients and reducing active LA [83]. Conventionally, the use of this practice produces several advantages as balanced grapevine shoot vigor, an amelioration of the canopy microclimate, as well as the facilitation in mechanized operation [96].

The veraison phase of Tempranillo was delayed by 20 days by making a severe cut immediately after fruit set. At the same harvest time, the fruits on the trimmed vines (LA/FW = 0.64) showed a soluble solids reduction (−3.5 °Brix) with respect to the control (LA/FW = 1.88); however, the treatment also significantly reduced both yield and total anthocyanin content [83]. Differently, late trimming in Sangiovese vineyard increased the total berry skin anthocyanin and phenolic concentration, without affecting the soluble solids, pH, and titratable acidity at harvest of berries, whereas it reduced yield, loosened bunches, and limited the severity of botrytis bunch rot [97].

In another study, the post-veraison ST of Sangiovese vines resulted in a reduction in must sugar concentration without altering the pH, the content of organic acid and anthocyanins, as well as the skin and seed tannins content [98]. Similar results for phenolic compounds were found in Sangiovese vines, in which the treatment did not significantly affect total and extractable anthocyanins, skin, and seed tannins [99].

Another study reported that LA reduction by trimming after berry set (berry diameter 3–4 mm) delayed the ripening and reduced sugar levels, with a positive effect on anthocyanin content [100].

Furthermore, a study carried out for 3 years on Grenache showed that the shoot trimming resulted in a consistent delay in grape ripening (20 days), reduced pH values (0.1 to 0.3), lower content in soluble solids (21.0 vs. 24.4 °Brix), and a decrease (10% to 27%) in the levels of total anthocyanin [83].

Lately, severe ST (SST) has become a common viticultural practice to regulate the vines’ source/sink ratio, which, by its effect on delaying ripening, was also suggested as a strategy to cope with the negative effects of global warming [101]. However, some authors found that SST carried out in the early phases of berry development (late veraison) can be a limiting factor for the buildup of both sugars and anthocyanins [102]. On the other hand, the reduction in assimilation area and the relative reduced sugar accumulation by SST remains underinvestigated, and the limited data available in the literature appear discordant. It seems that early SST delays ripening, and reduces Brix and anthocyanins. By contrast, other studies have shown that manipulating different SST treatments could cause a reduction of 0.4–1.7 mg/berry/d (0.02–0.11 °Brix/d) in grape sugar accumulation without affecting the yield and can significantly increase flavonols concentration [103,104].

Thus, it would be extremely beneficial a canopy management that consider the climate trends in viticulture regions, in lieu of being preemptively applied. Therefore, future and in-depth studies on the relationships between climate and phenology of the vine could provide important information on the management of the vineyard canopy in order to decrease the concentration of grape sugars and the resulting wine alcohol content, without undermining the quality of both raw material and final product [83].

#### 2.1.2. Winter Pruning

Winter pruning is a valuable tool for the modulation of vine vigor and yield, in the attempt to obtain specific and desirable compound composition in must. In some regions, as the Mediterranean growing areas, this activity is generally performed in the period from leaf fall to budbreak.

It has been shown that spur pruning at the phenological stage of swollen buds can delay vegetative growth, flowering, fruit set, and ripening.

In New Zealand, pruning carried out on Merlot at a length of apical shoots on the canes of about 5 cm decreased the grape sugar concentration and increased their content in organic acid [67,68,69].

By comparing pruning dates (early May vs. February–March), it was shown that post-budburst spur-pruning resulted in a decrease in fruit set and berry weight, as well as a slower fruit ripening. In particular, in the case of treatment in May, the soluble solids of the must and the titratable acidity were 1.6 °Brix lower and 1.8 g/L higher, respectively, with respect to values obtained at the other pruning dates [105].

Double-pruning represents another viticultural practice resulting in forcing the bud growth in spring and summer, thus causing a shift in the ripening of the berry, which occurs in a cooler period. In this way, both phenolic composition and chromatic parameters of the berry result improved, and a better sugar/phenolic content can be achieved [106].

### 2.2. Pre-Harvest Irrigation

In general, irrigation is a common cultural practice in viticulture in the Earth’s Western Hemisphere countries, whereas in Europe, its use for wine production is still quite limited or even forbidden on the basis of a common consideration, not scientifically proven in many cases, that irrigation negatively affects the composition of wine [107,108].

As an example, several winemakers reported a significant grape ripening delay and reduced wine quality as a result of increased irrigation in the last few weeks before harvest.

Irrigating plants after veraison could result in decreasing grape sugar content, even more so if this practice were carried out in combination with ST treatment. ST and plant irrigation, in fact, stimulate lateral shoots to compete with berries for available nutrients. However, a dense vine canopy that derives from an abundant water supply can also produce a decrease in wine color due to excessive shading of clusters [109].

A study showed the potential of combined use of pruning and increased irrigation to delay ripening, although it is necessary to analyze the implications that the obtained delay had on lower values of anthocyanins and phenolics observed in pruned vines that were not solely due to delayed ripening [101]. It must be noted also that irrigation intensity may result in different effects, since a full dose irrigation may produce a ripening delay (and lower sugars), whereas the regulated deficit irrigation, at the same time, may even stimulate ripening and the accumulation of sugars. The literature, in fact, reports several contrasting pieces of evidence about the variations occurring in sugar content due to plant irrigation. In a three-year study, Tempranillo vines were irrigated at different regimes and evaluated for yield and grape composition. Samples from irrigated vines showed a significant increase in total soluble solids and concentrations of grape sugars (glucose and fructose) with respect to values from samples of non-irrigated vines [110]. Conversely, other reports suggest that the composition of both must and wine can be influenced by irrigation, but in a manner that strongly depends on the specific climate conditions occurring during production [111,112].

Therefore, with respect to different viticultural approaches, pre-harvest irrigation could not be the best choice, whether the winegrower’s goal is limited to delaying the ripening of the grapes.

### 2.3. Managing Harvest Date

The management of harvest date represents an additional pre-fermentation strategy useful to obtain wines with decreased alcohol content [113,114].

Grape ripening is a critical phenological phase during which many metabolites that impact wine quality accumulate in the berries [115,116].

The production of lower alcohol wine has been studied in some varieties by checking the influence of early harvesting of grapes, as well as the blending of grapes, must, or wines from unripe and fully mature grapes [49,114,117,118,119,120,121,122]. Unripe grapes were used to produce a low-sugar and high-acid mixing material to be successively added to the must made from ripe grapes; this method has been reported to result in a significant decrease in ethanol concentration (−3% *v*/*v*), although the wines exhibited undesirable acidic and herbaceous characters [48].

Other studies aimed at investigating the effect of blending musts from unripe grapes with musts from well-ripened grapes have been conducted on several *V. vinifera* varieties (Shiraz, Malbec, Cabernet Sauvignon, Grenache, Pinot noir, and Tannat); these mixed wines showed higher total acidity and lower alcohol and pH content [49,120,123,124].

By blending must of mature grape with juice from unripe grape, Piccardo and coauthors obtained wines with a lower pH and alcohol content (14–21% reduction range with respect to control wines values), whereas wine color intensity was increased, as well as the concentrations of phenolic compounds and anthocyanins, proanthocyanidins, and polysaccharides content [49].

Double harvesting (at lower and higher maturities of the grape) has been proposed as an alternative approach for decreasing must sugar concentration. As carried out in a study in 2013, grapes harvested at low maturity (15.2 and 13.4 °Brix) were used together with another batch in which the harvesting was carried out when high phenolic maturity and high sugar levels were reached (>24 °Brix) for the production of the same wine [83].

### 2.4. Other Techniques

In addition to the previously described strategies (defoliation, trimming, and pruning) classically applied in canopy management, other techniques can be used to indirectly affect grape sugar accumulation. These involve the use of shading nets, anti-transpirant products, and growth regulators.

#### 2.4.1. Shading Nets

Another possible way to reduce the accumulation of sugars in grapes is based on the use of shade nets. These sheets are placed to cover different parts of the canopy to diminish the solar radiation that reaches the leaves, thus inducing a reduction in photosynthesis. This reduction in photosynthetic activity can improve water use efficiency and slow down the ripening process, preserving must acidity, sensory quality, and volatile flavors [125,126].

By applying post-veraison a white cloth above the canopy (62% absorption), at harvest, Shiraz grapevines showed reduced values of both pH and total soluble solids (−1.5 °Brix) and increased titratable acidity [50].

In another study, the timely use of shading (30–50% of the direct sunlight) on Syrah vines (berries of 5 mm) resulted in a general delay in grape ripening, causing a significant reduction in sugar concentrations [127].

By covering Cabernet franc vines with a bird net (50% light interception), a grape must with a significant reduction in soluble solids content and pH was obtained [128].

Lastly, Alphones Lavallée and Narince grapes shaded at veraison by black nets showed berries with increased total soluble solids [129].

Therefore, the use of artificial shading can be a valid alternative in viticulture to slow down the ripening process.

#### 2.4.2. Anti-Transpirant Products

A different technique to reduce berry sugar accumulation without physical reduction in leaf area consists of anti-transpirant products applied to form a thin layer. Once sprayed over the canopy, these compounds polymerize, creating a film layer which results in an anti-transpirant effect [109].

As an example, a study demonstrated that total soluble solids of Cabernet Sauvignon grapes were reduced by 2.09 °Brix after treatment with the film-forming anti-transpirant agent 1-*p*-menthene (also known as pinolene) [51].

Advantageous anti-transpirant action of pinolene was also shown in another study in which its application to Falanghina vines induced a significant reduction in the rate of net photosynthesis (25–40%) and stomatal conductance (40–60%) on the leaves, a reduced level of sugars in the berries (2 °Brix), resulting in wine with lower alcohol content (0.9–1.6% *v*/*v*) [130].

Similarly, the application of a pinolene-based natural anti-transpirant on post-veraison Sangiovese grapes (2% concentration to the upper 2/3 of the canopy) proved to be efficient in decreasing the grape ripening (−1.2 °Brix at harvest), as well as the ethanol level (−1% *v*/*v*) in the obtained wine [52].

Pre-veraison use of pinolene either alone or in combination with a pre-flowering application has been shown to be effective in delaying sugar accumulation in Barbera grapes (−2.4 and −3.7 °Brix, respectively, vs. control) without affecting color development [131].

Similar results were obtained in another recent study on Sauvignon Blanc vines in which the use of pinolene significantly reduced the concentration of sugar in the berries at harvest by −1.5 °Brix compared to the control [132]. The effectiveness of this compound on Sauvignon Blanc vines was also shown in a previous study in which the anti-transpirant effect has been evaluated in three consecutive years: at harvest, a significant difference between pinolene-treated and untreated grapes was found (average values −2.09 °Bx) resulting in reduced alcohol content (−1.06% *v*/*v*) in wine [53].

In a recent study, the efficacy of fulvic acid as an anti-transpirant was checked on both Cabernet Sauvignon and Riesling ripe grapes: the total soluble solids of grapes significantly decreased for Cabernet by 0.6 °Brix and 1.1 °Brix in 2017 and 2018, respectively, and for Riesling grapes by 1.5 °Brix and 1.0 °Brix, for the same period [133].

#### 2.4.3. Application of Growth Regulators

Exogenous application of growth regulators to either the bunch zone or the whole canopy may be a useful tool for delaying the onset of sugar ripening [134].

Over the last few decades, a progressive reduction in the employment of plant growth regulators has occurred. This is mostly due to several factors, among which the principal ones are (i) increasing legislative restrictions about the use of chemicals, (ii) the incomplete knowledge of sophisticated mechanisms behind physiology regulation, (iii) the variability of produced effects. In viticultural practice, in fact, improper or excessive use of growth regulators can lead to abnormal plant growth, and some compounds can have toxic effects on humans or animals if ingested. In particular, although synthetic auxins can be useful at certain concentrations, if used at high doses, they can be phytotoxic, causing growth abnormalities, tissue damage, and even death in plants [135]. In addition, residues of these synthetic compounds in agricultural products can be harmful to animal and human health due to their toxicity, and for this reason, their use is subject to strict regulations due to the potential risks to the environment and health [136,137,138]. Complying with these regulations can increase costs and limit market growth [139]. Thus, natural auxins are often preferred for their eco-friendly properties; however, their production costs are typically higher than synthetic alternatives, hindering their widespread adoption [140,141].

Despite that, for some of these compounds, there is a novel interest in the light of their capability to interact with the ripening process, and, in particular, to induce slower ripening. In particular, auxin, and especially its synthetic analogs 1-naphthalene acetic acid, has been used to obtain a delay in ripening. Böttcher and coauthors, in fact, treated Syrah grapes in pre-veraison with the anti-transpirant 1-naphthalene acetic acid, thus obtaining an effective delay of berry ripening and a better sugar level control, together with the absence of negative effects on wine sensory properties [142].

In another study, Shiraz and Cabernet Sauvignon grapes treated in pre-veraison with the same compound showed a 3–4-week delay in reaching harvest maturity [143].

Application of this compound could represent a useful manner for delaying grape ripening, as well as for extending the harvest period. More research is still needed to ascertain the pathways responsible of the auxin effect in delay ripening, however it has been proposed that auxin application could preserve berries in the pre-veraison state, as suggested by the lateness of canonical biochemical changes (increases in both sugars and anthocyanins, reduced acidity, and lower chlorophyll levels) observed in ripening [144,145].

In summary, the use of some cultivation techniques has a direct or indirect impact on the accumulation of sugar in the berries; however, it is not always possible to achieve a drastic sugar reduction without compromising other grape metabolites such as polyphenols and aromatic compounds [41]. Moreover, it must be noted that the variability of the experimental results obtained in the above-mentioned studies derives from the complex interaction between grape variety, rootstock, and environment. This interaction makes it difficult to draw definitive conclusions about the effectiveness of each individual viticultural method adopted to limit the accumulation of sugars in grapes. It is easy to suppose that the best results could derive from optimizing the application of several viticultural techniques at the same time.

## 3. Pre-Fermentative Strategies

As for viticultural techniques, pre-fermentation strategies aimed at decreasing ethanol production are also based on the reduction in fermentable sugar content, which is primarily achieved by filtration and/or dilution of grape must. As an additional biotechnological strategy, some studies proposed as alternative tool the use of enzymes (mainly Glucose Oxidase/Catalase) supplementation. All these approaches were discussed below (and summarized in Table 1).

### 3.1. Filtration of Grape Must

**Figure 1:**
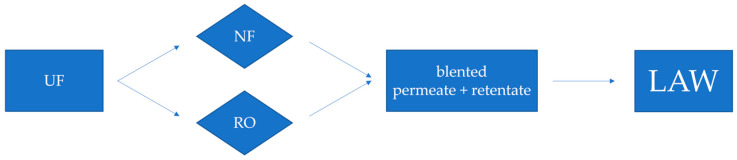
Scheme of grape must filtration for the production of wines with reduced ethanol content. Abbreviations: UF, ultrafiltration; NF, nanofiltration; RO, reverse osmosis; LAW, low-alcohol wine.

Membrane filtration has been used in wine production for a long time; classical examples are the ultrafiltration used to clarify white wine from grape must, as well as the nanofiltration (NF) and reverse osmosis (RO) applied for sugar concentration in musts (Figure 1) [146,147].

However, several studies have proposed membrane filtration of grape must juice as a method to produce wines with a low alcohol content, by taking advantage of the reduction in sugar content of must before fermentation [61,63,148]. Such a process consists of extracting the sugar from musts by using membrane coupling, combining microfiltration or ultrafiltration with NO or RO [112,113]. After that, filtered juice and the one rich in sugar are added, and fermentation can start [25]. In greater detail, in the NF technique, by means of a pressure gradient, a portion of must is forced to pass through a membrane, thus separating two fractions, namely permeate and retentate, which are characterized by a lower and higher sugar concentration, respectively. After this stage, a must showing the desired levels of sugar can be obtained by combining in appropriate proportions the two fractions (Figure 1) [149].

NF was successfully applied to both red and white musts to produce wines with reduced alcohol content. A study carried out on Verdejo and Tinta de Toro grapes showed that mixing, in adequate proportion, untreated musts with the permeate and retentate obtained from the first NF stage resulted in white and red wines with alcohol content reduced by about 3% and 2% (*v*/*v*), respectively [61].

Salgado and coauthors applied a single-stage and two-stage NF process (200 Da spiral-bound membrane) to both white and red musts. Compared to the control (wine obtained by untreated musts), wine resulting from the fermentation of properly mixed filtration permeate and retentate showed a marked decrease (1–2% *v*/*v*) in ethanol content, but preserved their original sensory qualities [62].

It has been found that filtration techniques, while having an important impact on reducing the sugar levels of the must, can cause phenomena of retention of aromatic compounds precursors [150,151]. Thus, the choice of the best technical conditions, as well as the appropriate selection of molecular weight for membrane cut-off, could increase the retention of volatile compounds and preserve the good taste in wine [151,152].

Another pre-fermentative technique to reduce must sugar concentration is RO, which consists of applying a pressure higher than the osmotic one, in order to force low-molecular-weight compounds (water, alcohol, etc.) to pass through a semi-permeable membrane, whereas tannins and other sensory compounds are retained [27,63]. Also in this case, for production of wine with a lower alcohol content (≤10.5% *v*/*v*), optimal operating conditions and the right membrane configuration (molecular weight cut-off) must be taken into account to avoid the leak of valuable organic compounds, as polyphenols and anthocyanins, thus affecting both color intensity and sensory properties [29,153].

RO has been used on musts of several grape varieties, obtaining the two fractions: one poor in sugar (permeate) and the other richer in sugar (retentate); these have been successively blended in appropriate proportions and wines with an ethanol content of 5%, 7%, 10% and 13% (*v*/*v*) were obtained. However, besides the decreased alcohol, polyphenol levels were also found to be reduced, which affects the sensory properties of the wines [63].

In addition, it must be noted that RO involves the removal of ethanol together with water; thus, the further necessary step after filtration is water re-addition to the low alcohol product, which raises legal concerns in those countries in which this practice (water supplementation) is prohibited (see below) [154]. In addition, grape juice filtration, particularly with techniques such as RO, can be energy-intensive due to the high pressures and temperatures often required to separate the juice components [155,156].

### 3.2. Addition of Enzymes

**Figure 2:**
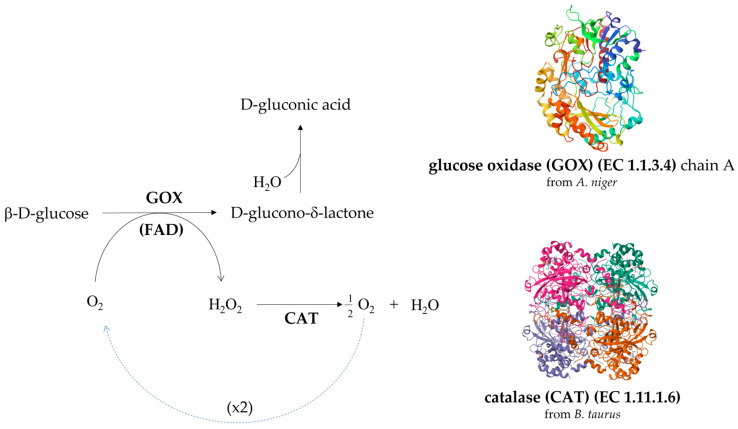
Biochemical reactions (**left**) and molecular structures (**right**) of glucose oxidase (GOX) and catalase (CAT). The reported structures refer to enzymes from *Aspergillus niger* (GOX, PBD: https://doi.org/10.2210/pdb1cf3/pdb) and *Bos taurus* (CAT, PBD:  https://doi.org/10.2210/pdb4BLC/pdb), whose use has been approved by FDA.

The production of wines with lower alcohol content can also take advantage of the activity of enzymes, such as glucose oxidase (GOX) and catalase (CAT). This enzymatic method can be used to reduce grape must glucose before alcoholic fermentation and leads to must acidification, in addition to the reduction in sugar concentration [157,158]. GOX (EC 1.1.3.4) is a conjugated protein, whose prosthetic group is the flavin adenine dinucleotide (FAD), falling in the oxidoreductases class (EC 1), acting on the CH-OH group of donors (EC 1.1). This flavoenzyme catalyzes the oxidation of β-D-glucose to D-gluconic acid δ-lactone; being an oxidase (EC 1.1.3), the enzyme transfers by its FAD the reducing equivalents directly to molecular oxygen, which is reduced to hydrogen peroxide (H_2_O_2_). In a second step reaction, D-gluconic acid δ-lactone spontaneously hydrates to form gluconic acid, and this process lowers must pH [159]. The second enzyme, CAT (EC 1.11.1.6), is required to convert H_2_O_2_ into water and (again) oxygen that returns as substrate for GOX reaction (see for stoichiometry Figure 2). Many commercial kits offer GOX added with CAT, since it is important to quickly remove H_2_0_2_; the accumulation of this product, in fact, would result in GOX inactivation, as well as potential oxidation of valuable compounds, and inhibition of fermentative yeasts proliferation.

Several studies have reported the use of GOX for the reduction of wine alcohol [56,160,161]. It is important to note that the U.S. Food and Drug Administration (FDA) classified GOX and CAT as GRAS (Generally Recognized As Safe), and the use of both enzymes (whose structures and sources are shown in Figure 2) in wine production has been authorized [162]. The OIV does not provide a specific regulation about the application of GOX and CAT; however, enzymes addition in wine production is generally allowed if their use respects the mandatory condition to not represent a risk for health and/or for wine quality [163].

In 1998, Pickering and coauthors firstly applied the GOX enzyme method on Riesling and Müller–Thurgau white grape juices to obtain wines with a lower alcohol content. By using Ca_2_CO_3_ addition, the must pH was increased to optimize GOX reaction, thus obtaining more than 40% increase in final gluconic acid concentration, even though the treatment somehow affected wine organoleptic properties [160].

Later on, the same authors used 2 g/L of GOX in Riesling grape juice, obtaining the conversion of about 87% glucose into gluconic acid (after 6 h of fermentation), resulting in a decrease in alcohol content up to 4.3% *v/v* [55].

In another study, a GOX product obtained from *Aspergillus oryzae* was added to Pinotage grape must before fermentation. Compared to control, wines obtained from treated must showed a 0.7% *v/v* decrease in ethanol content [56].

However, these studies showed that the wort acidity (low pH values) represents the limiting factor for the activities of both enzymes.

In this regard, more recent studies have shown an improvement of both GOX and CAT efficiency, whether they were encapsulated in Ca-alginate hydrogel, thus suggesting this strategy as a valid tool to be used in low-alcohol wine production [164].

More recently, co-immobilization of both enzymes in silica-Ca-alginate hydrogel showed a remarkable ability to reduce glucose level in must, combined with a decreased release of gluconic acid. As a result of encapsulated enzymes treatment, a noteworthy glucose consumption (up to 26 g/L) led to a reduction in the potential must ethanol content (−2.0% *v*/*v*). In addition, a significant decrease (74%) in the estimated concentration of gluconic acid was obtained [57].

It must be noted that the treatment of musts with GOX could also result in lower concentrations of several phenolic compounds and some volatile organic compounds (VOCs) responsible for floral notes in the obtained wines.

Treatment of a white grape must with GOX enzyme gives musts with a marked decreased in alcohol content at pH 3.4–3.5, which resulted in more acidic (2.7–2.8) and less fruity wines [157].

In a recent study, the enzymatic treatment with GOX and CAT was applied to Verdejo must, causing a notable decrease in glucose levels (61.5 g/L) and a reduction in alcohol volume (2–3% *v*/*v*) in the produced wine. However, concentrations of some VOCs responsible for floral and fruit notes (heptyl acetate and 2-phenylethanol, ketones) were lower in wines from treated musts. Moreover, the treatment of musts also resulted in a significant reduction in other important compounds (total polyphenols, flavonoids, and hydroxycinnamic acid) [58].

Conversely, it was reported that the addition of GOX in combination with CAT decreased the pH of the must, reduced wine alcohol concentration, and had a positive impact on the sensory profiles of Tempranillo wine [165].

### 3.3. Grape Must Dilution

To reduce ethanol yield, a further pre-fermentation technological approach available to winemakers is represented by either the addition of (dilution) or substitution with (“bleed and replace”) water to high sugar musts before fermentation [166].

Nevertheless, the majority of countries have severe regulations about the use of water in wine production. Generally, the addition of water to either musts or wines is not allowed or, in some cases, strictly regulated by competent authorities. OIV restricts the practice of water addition in winemaking to the cases of “aromatized wines”, “beverages based on vitivinicultural products”, and “wine-based beverages” [167]. The unique exception is represented by the reintroduction of must in water and organic acids previously nanofiltrated by membranes with the aim to reduce must sugar concentration [168].

In the European Union (EU) wine regulations, water addition is not allowed in wine production “except where required on account of a specific technical necessity” (Regulation No 1308/2013) [18].

Conversely, as a tool to facilitate fermentation, the addition of water to wine is authorized and commonly accepted by the USA legislation [169].

The FSANZ (Food Standards Australia New Zealand) allowed for adjusting must sugar concentrations by water addition (before yeast addition to start fermentation) with the double aim of ensuring a correct fermentation and to mitigate wine ethanol content. Anyway, water addition cannot dilute the sugar level of grape must below the value of 13.5 degrees Baumé (Bé) [170].

In addition, it must be noted that must dilution either by water addition or by blending juices obtained from less mature grapes, surely result in a reduction in fermentable sugars, but, at the same time, also produces side-effects, as a reduced must acidity and in some cases a detrimental impact on physical and sensory properties of the wine [171].

For instance, Piccardo and coauthors showed that for *V. vinifera* cv. Merlot Noir and cv. Tempranillo Tinto, water treatments reduced ethanol levels, but resulted in significant differences in flavor profiles for each variety [49].

In a recent study, the direct addition of water to the must in winemaking in late harvests (15.5 °Bé) and medium harvests (14.5 °Bé) produced wines with lower alcohol levels while avoiding the unfavorable unripe “green” chemical and sensory attributes that can be present in wines produced from early harvests [172].

In another study, it was reported that water substitution treatments could be a valid strategy to control alcohol levels in Cabernet Sauvignon and Shiraz wines without any critical effect on wine color and with limited variations in volatile compounds and sensory profile [121].

The same research group also demonstrated that water substitution treatments (at varying levels) of a late harvest (15.4 °Bé) high sugar must were able to decrease the alcohol levels in the Shiraz wines by 0.5–2.0% *v/v* compared to the control wine [59].

Accordingly, in another study, musts from late harvest (13.5 °Bé) treated with water showed a reduction in wine ethanol from 1.6% (*v*/*v*) to 2.1% *v/v* with respect to wine obtained by not-treated musts, without a negative impact on phenols, tannin composition, and color properties of wine [60].

Therefore, although these results suggest that the addition of water can be considered an easy strategy to control wine alcohol content, further research is still required to better investigate the effects of such a practice on both volatile profile and sensory attributes of the produced wine.

## 4. Conclusions

The increasing interest of consumers in beverages with reduced alcohol content pushes the wine industry to manage the growing demand, as well as to take into account the modified scenario in this strategic sector.

This requires a multi-pronged approach. In the vineyard, for example, wine grape growers can reduce leaf areas, irrigate vines just before harvest, apply growth regulators in the vineyard, and also review and optimize the harvest date.

In the winery, winemakers have several pre-fermentative approaches available to them to produce wines with lower, more favorable alcohol levels: dilution of the must, combined use of GOX-CAT enzymes, and specific filtration techniques (nanofiltration and reverse osmosis). In addition, current technologies also allow for the reduction in ethanol levels in post-fermented wine. However, pursuing the goal by a proper selection of viticultural and pre-fermentation strategies appears to be a successful approach.

## Figures and Tables

**Table 1:** Overview of alcohol reduction in wines obtained by the application of different viticultural (in white) and pre-fermentative (in gray) strategies.

Strategy	Techniques (Grape Cv./Wine)	Alcohol Reduction (*v*/*v*)	Ref.
defoliation	post veraison leaf removal (Sangiovese)	0.6%	[43]
	apical defoliation (Shiraz)	0.2–0.7%	[44]
	pre-flowering defoliation (Trnjak)	0.2%	[45]
pruning	pruning severity modulation (Malbec)	0.7%	[46]
	shoot trimming (Grenache, Tempranillo)	2%	[47]
harvest date management	unripe grapes—cluster thinning (Grenache)	3%	[48]
	unripe grapes (Pinot and Tannat)	0.5–3%	[49]
shade	overhead shade (Shiraz)	1%	[50]
anti-transpirant agent	pinolene application (Falanghina)	0.9–1.6%	[51]
	pinolene application (Sangiovese)	1.0%	[52]
	pinolene application (Sauvignon)	1.0%	[53]
enzyme addition	GOX ^1^ (Muscat-Ottonel)	1.05%	[54]
	GOX (Riesling)	4.3%	[55]
	GOX preparation from *A oryzae* (Pinotage)	0.7%	[56]
	encapsulated GOX-CAT (Verdejo)	2.0%	[57]
	GOX-CAT (Verdejo)	2–3%	[58]
must dilution	late harvest (Shiraz)	0.5–2.0%	[59]
	three stages harvesting (Shiraz)	1.6–2.1%	[60]
filtration and membrane processing	must NF (Verdejo and Tinta de Toro)	2–3%	[61]
	must NF (Verdejo and Garnacha)	1–2%	[62]
	must RO (Tinta Roriz, Syrah, Alicante Bouschet)	1.5–10%	[63]

^1^ Abbreviations: GOX, glucose oxidase; *A. oryzae*, *Aspergillus oryzae*; CAT, catalase; NF, nanofiltration; RO, reverse osmosis.

## Data Availability

The original contributions presented in the study are included in the article, and further inquiries can be directed to the corresponding authors.
